# Chalcones Repressed the AURKA and MDR Proteins Involved in Metastasis and Multiple Drug Resistance in Breast Cancer Cell Lines

**DOI:** 10.3390/molecules23082018

**Published:** 2018-08-13

**Authors:** Tatiana Takahasi Komoto, Tayná Minervina Bernardes, Thaís Balthazar Mesquita, Luis Felipe Buso Bortolotto, Gabriel Silva, Tamires Aparecida Bitencourt, Seung Joon Baek, Mozart Marins, Ana Lúcia Fachin

**Affiliations:** 1 Biotechnology Unit, University of Ribeirão Preto, SP, Av. Costábile Romano, 2201, Ribeirão Preto, SP, CEP 14096-900, Brazil; tattytk@hotmail.com (T.T.K.); taynalink@yahoo.com.br (T.M.B.); thaismesquita26@hotmail.com (T.B.M.); lfbortolotto@gmail.com (L.F.B.B.); biel-189@hotmail.com (G.S.); tabitencourt@yahoo.com.br (T.A.B.); mmarins@gmb.bio.br (M.M.); 2 Department of Biomedical and Diagnostic Sciences, College of Veterinary Medicine, University of Tennessee, Knoxville, TN 37996, USA; sbaek2@utk.edu

**Keywords:** flavonoids, BT-20, MCF-7, metastasis, MDR

## Abstract

In the present investigation, *trans*-chalcone and licochalcone A were tested against MCF-7 and BT-20 breast cancer cell lines for anti-tumor activity. We found that both chalcones down regulated important genes associated to cancer development and inhibited cell migration of metastatic cells (BT-20). Finally, we observed that licochalcone A reduces the MDR-1 protein, while both chalcones suppress the AURKA protein in a dose-dependent manner. In conclusion, we observed the *trans*-chalcone and licochalcone A affected the cell viability of breast cancer cell lines MCF-7 and BT-20 and presents anti-metastatic and anti-resistance potential, by the repression of AUKA and MDR-1 proteins.

## 1. Introduction

Cancer is characterized by uncontrolled cell proliferation, which results from accumulated mutations in the DNA of the cells. Among different types of cancer, breast cancer is the most common cancer in women worldwide, contributing nearly 29% of all cases and showing high mortality rates [1,2,3].

Metastasis is characterized by the ability of cancer cells to invade adjacent or distant tissues, developing secondary tumors. It is responsible for about 90% of cancer-related deaths. The main sites of breast cancer metastasis are the brain, bones, kidneys and lung [4,5]. Although metastasis is accountable for the high mortality rates in cancer patients, this process has not yet been completely elucidated. However, it is known that some genes are related to this process, such as the Aurora Kinase A gene (AURKA). The AURKA plays a role as a regulator of cellular mobility in breast cancer [5], therefore, drugs that act on this target may decrease metastasis.

After the advent of breast cancer treatments such as chemotherapy, local control surgery and radiotherapy [6], the survival rate of cancer patients has increased steadily over recent years, however, such treatments still have many side effects. In addition, tumors have shown resistance to chemotherapy due to the over-expression of ATP-binding cassette superfamily (ABC) transporter genes [7], which pump the chemotherapeutic out of the tumor cell [8]. Among the ABC family genes, the MDR-1 (G-glycoprotein-P) and ABCG2 genes are associated with drug resistance in breast cancer [9].

Thus, new drugs with fewer side effects, greater efficiency, and specificity for the tumor cell are needed for breast cancer treatment. In this context, among natural small molecules, chalcones have gained attention due to their ability to regulate cancer-related molecular pathways, such as apoptotic pathway, metastasis, and cellular stress response [10,11,12].

In this article, *trans*-chalcone and Licochalcone A differentially modulate gene expression in BT-20 (triple-negative) and MCF-7 (responsive hormone) breast cancer, as well as reduced levels of MDR1 and AURKA proteins.

## 2. Results

### 2.1. Cytotoxicity of Chalcones

**Figure 1:**
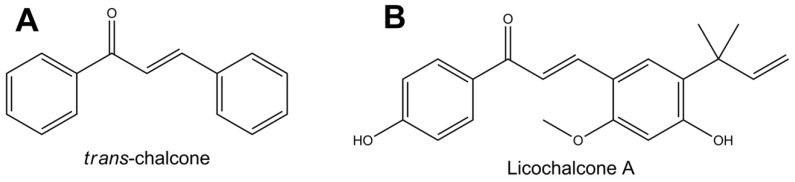
Structures of the two chalcones tested.

Cytotoxicity of *trans*-chalcone and Licochalcone A (Figure 1) was evaluated against MCF-7 and BT-20 breast cancer cells after exposure for 24 h. Chalcones showed pronounced inhibitory activity for both cell lines, however, a higher sensitivity was observed for the BT-20 cells (Table 1).

### 2.2. Trans-Chalcone and Licochalcone A Modulate Genes Involved in Drug Resistance and Metastasis in MCF-7 Cell

**Figure 2:**
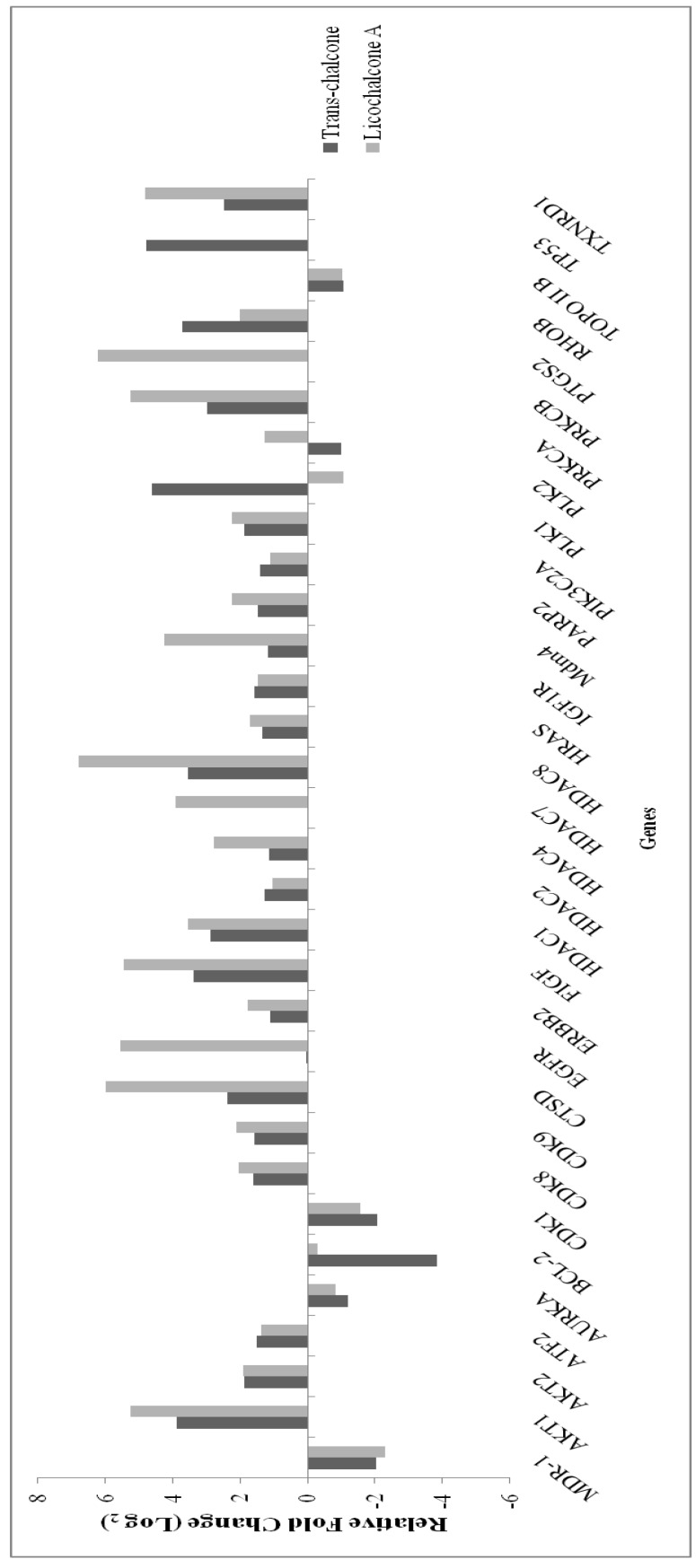
Profile of gene expression by PCR array for MCF-7 cell line.

**Figure 3:**
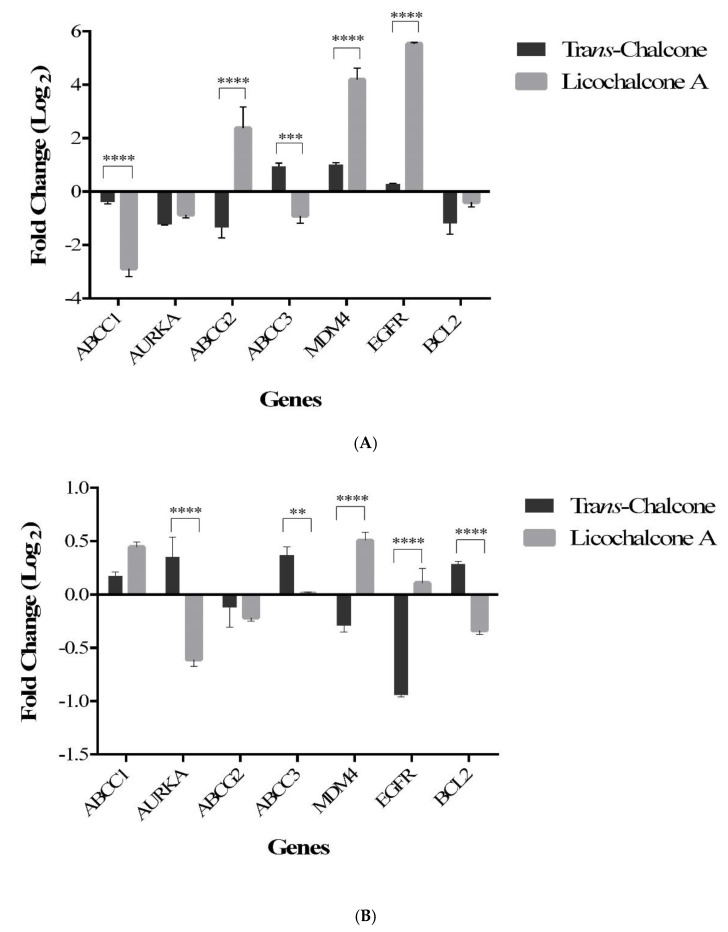
Analysis of the transcriptional profile by qRT-PCR in (**A**) MCF-7 cell line and (**B**) BT-20 cell line. The data represent the mean ± SD of three experiments. ** *p* ≤ 0.01, *** *p* ≤ 0.001 and **** *p* ≤ 0.0001. The asterisks represent significant difference between chalcones.

The PCR Array Human Cancer Drug Targets profiles the expression of 84 genes considered targets for anticancer therapeutics and drug development. The treatment of cells with chalcones resulted in the significant modulation of 32 genes (Figure 2). Among these, 7 genes were analyzed by qRT-PCR in the MCF-7 and BT-20 cells in order to better evaluate the effect of chalcones in some important cancer-related pathways, such as multiple drug resistance (ABCC1, ABCC3 and ABCG2), apoptosis and cell cycle (Bcl-2 and MDM4), growth factor (EGFR) and metastasis (AURKA). RT-PCR analysis in MCF-7 cells showed that *trans*-chalcone and licochalcone A repressed the ABCC1, AURKA, and Bcl-2 genes, *trans*-chalcone repressed the ABCG2 gene, while licochalcone A suppressed the ABCC3 gene (Figure 3A). On the other hand, in BT-20 cells, both chalcones suppressed the ABCG2 gene, *trans*-chalcone repressed the MDM4 and EGFR genes, while licochalcone A repressed the AURKA and Bcl-2 genes (Figure 3B). 

### 2.3. Effect of Chalcones on Cell Migration and Invasion

**Figure 4:**
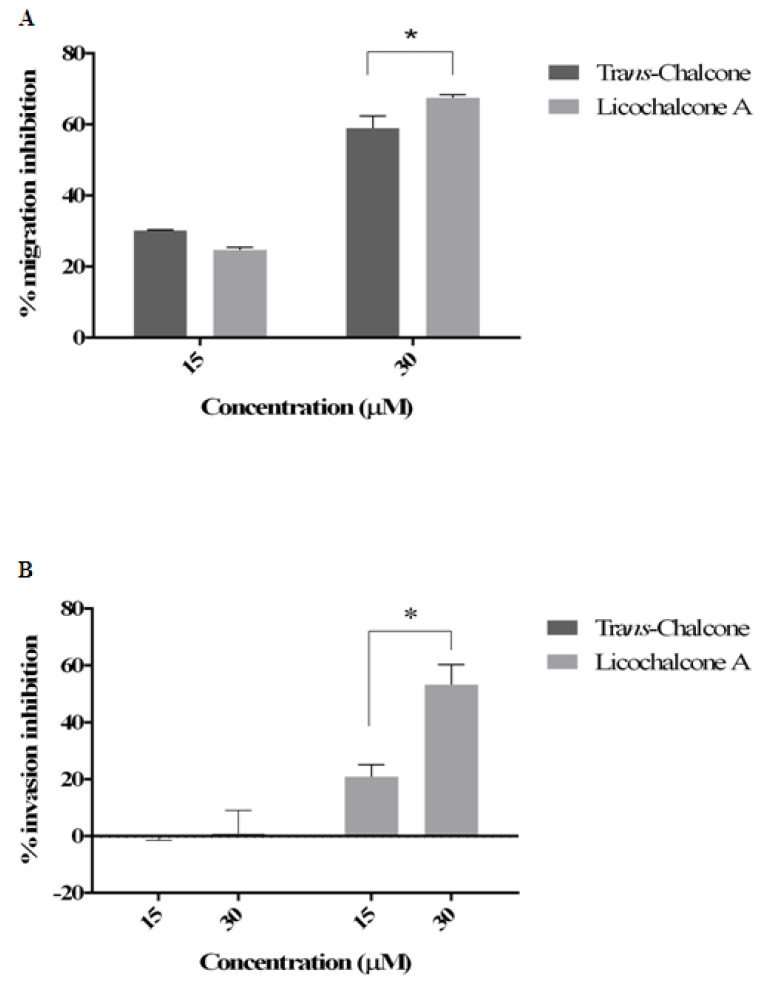
*trans*-chalcone e Licochalcone A inhibited cell migration and invasion in BT-20 cells. (**A**) Migration Inhibition (%) and (**B**) Invasion Inhibition (%) both experiments were calculated and quantitative results are illustrates bellow. The data represent the mean ± SD of three experiments. * *p* ≤ 0.05.

According to the data analysis, both chalcones reduced migration of BT-20 cells in a dose-dependent manner, with significant reduction at 30 µM to licochalcone A and *trans*-chalcone in 67.47% and 58.99% respectively, after 24 h (Figure 4A). However, only licochalcone A inhibited cell invasion in 53.33% (Figure 4B).

### 2.4. The Chalcones Act in AURKA and MDR-1 Protein

**Figure 5:**
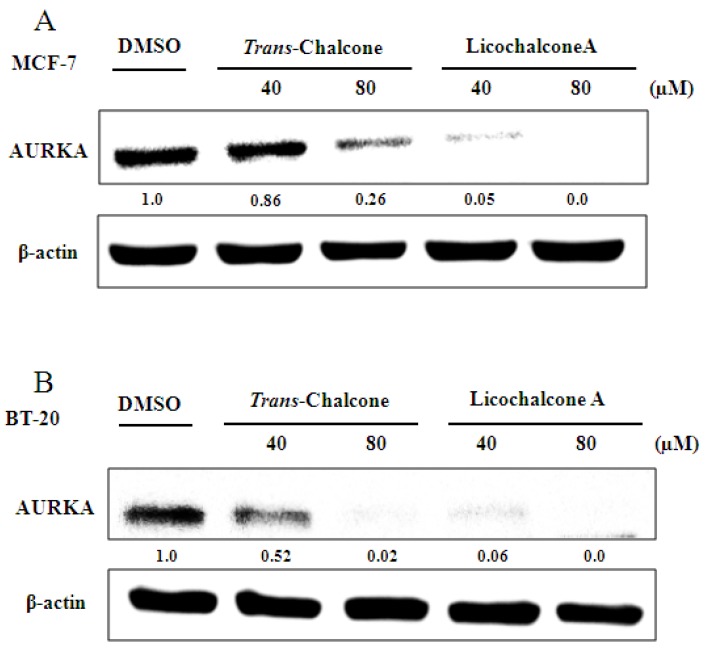
Analysis of chalcones effects at the protein level by western blot for AURKA. (**A**) Expression of AURKA protein in the MCF-7 Cell for both chalcones at different doses; (**B**) Expression of AURKA protein in the BT-20 Cell for both chalcones at different doses.

**Figure 6:**
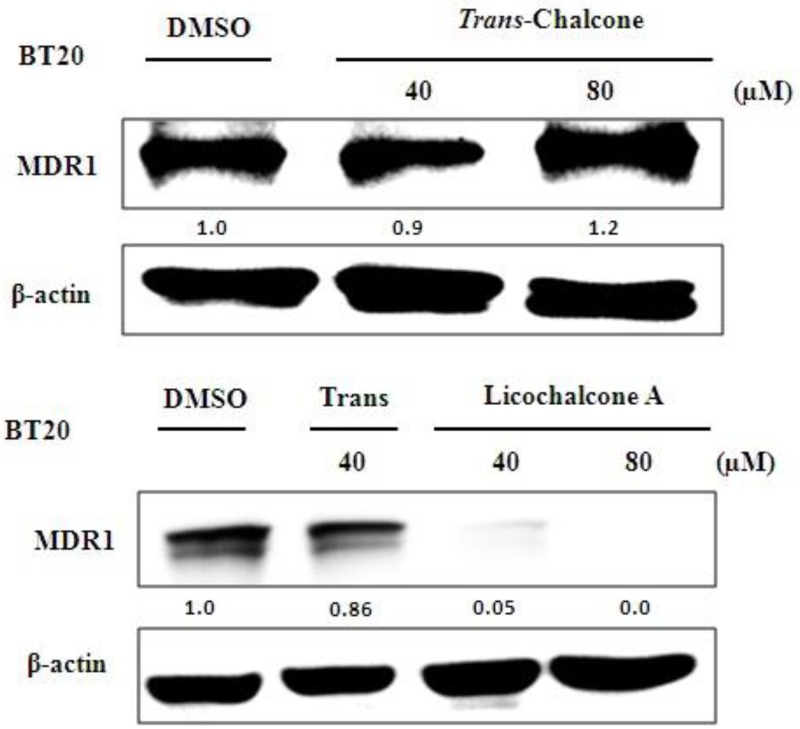
Western blot assays for expression of the proteins MDR-1 on BT-20 breast cancer cell line through the treatments of the different concentrations of *trans*-chalcone and licochalcone A for 24 h.

In the Western blot assays, it was observed that both chalcones inhibited the expression of AURKA protein in MCF-7 (Figure 5A) and BT-20 cells (Figure 5B) in a dose-dependent manner. However, only licochalcone A repressed the MDR-1 protein expression in the BT-20 cell line (Figure 6). We did not observe the effect of chalcones on MDR1 protein in MCF-7 because this cell line did not present basal expression of the MDR-1 protein in culture medium (data not shown).

## 3. Discussion

Breast cancer can be classified according to histological type, tumor grade, lymph node status, and presence of receptors such as ER (estrogen receptor), PR (progesterone receptor), and of the HER2 protein. This classification divides breast cancer into five main molecular subtypes: Luminal A, Luminal B, Basal-like, HER2-enriched, and Claudin-low [13]. Because of these characteristics, each subtype has different prognosis and treatments, so responsive hormone cells (Luminal A and B) respond well to hormone therapy. However, basal-like and claudin-low subtypes present difficult treatment, because they do not have therapeutic targets, making them more aggressive and with a worse prognosis [14].

The viability assay showed a dose-dependent manner for both MCF-7 and BT-20 cell lines to treatment with *trans*-chalcone and licochalcone A. These experiments allowed us to establish the IC_50_ for both chalcones. The commercial drug doxorubicin, normally used in cancer treatment, presents lower IC_50_ than chalconas [10], but shows serious side effects related with higher DNA damage induced by a doxorubicin such as cardiotoxicity [15]. Bortolotto et al. (2016) have demonstrated that both chalcones were cytotoxic for the 3T3 cell line (normal embryonic mouse fibroblasts), but this cytotoxicity was lower than for the MCF-7 cell line. Moreover, the DNA damage induced by both chalcones was lower than the doxorubicin-induced damage mainly in normal cells 3T3. These chalcones demonstrated antiproliferative activity against various types of cancer cell lines including breast cancer, osteosarcoma, and cancer gastric [10,16,17].

Analysis of gene expression using PCR array demonstrated that *trans*-chalcone and Licochalcone A repressed most genes related to drug resistance, tumor progression, and metastasis in MCF-7 (hormone-responsive luminal A) and also in BT-20 cell line (basal-like triple-negative) that presents a worse prognoses.

*trans*-chalcone suppressed the MDM4 gene expression in BT-20 cell line. This gene belongs to the family called Mouse Double Minute (MDM), responsible for regulating the stress response and cellular recovery. Normal expression of MDM4 is vital for normal breast development and preservation of chromosomal fidelity. However, increased expression of this gene is associated with the onset of tumors. Thus, MDM4 is active in 40% of breast cancers, showing that this gene can be considered a new target for antitumor therapies [11,18].

The chalcones down-regulated the Bcl-2 gene in MCF-7 cell, however, only Licochalcone A down-regulated the expression of Bcl-2 in BT-20 cell. In previous studies, *trans*-chalcone, and Licochalcone A also inhibited the Bcl-2 gene, and further induced apoptosis mediated by p53 induction in MCF-7 [10]. In addition, *trans*-chalcone showed activity in osteosarcoma lines acting on the modulation of p53 and Sp1 at post-translational level, inhibiting cell growth in a dose- and time-dependent manner [16]. Therefore, chalcones are indicated as regulators of the apoptotic and cell cycle pathways in several tumor types.

The AURKA gene is a proto-oncogene often overexpressed in malignant breast tumors, and it is involved in the genetic pathway underlying the origin of aneuploidy and loss of centrosome duplication control [19]. Furthermore, AURKA plays a role in cell cycle, apoptosis and metastasis [12,20,21,22]. High expression of AURKA is found frequently in several human malignancies, including breast and ovarian cancer. Its overexpression alters transcription and/or post-translational regulation, enhancing kinase expression in human tumors [23]. In addition, it was reported that BT549, MCF-7, MCF-10A, and MDA-MB-231 breast cancer cells when treated with AURKA inhibitor (ZM447439) showed low metastasis potential mediated by the inhibition of cell migration [5]. Thus, studies have indicated AURKA as an important target in breast cancer therapy [5,24].

Due to the repression of AURKA at transcriptional and post-translational levels induced by *trans*-chalcone and Licochalcone A, it was essential to evaluate the effect of the compounds on the migration and invasion of BT-20 cell line, which is characterized by high metastatic potential. Both chalcones at the concentration of 30 μM significantly inhibited the migration and invasion of BT-20 cells. This data suggests that chalcones may present anti-metastatic activity, which is relevant since metastasis is the leading cause of cancer-related deaths [25,26].

The multiple drug resistance (MDR) is one of the main problems of cancer treatment. Consequently, the knowledge of drugs that are not transported by MDR proteins is important as long as they decrease the chances of chemotherapy resistance [27]. Herein, this research shows that chalcones down-regulate genes involved in multiple drug resistance (MDR-1 e ABCG2) in MCF-7 and BT-20 cell lines.

## 4. Conclusions

Based on the data, it can be concluded that *trans*-chalcone and Licochalcone A reduce cell viability, migration, and invasion in breast cancer cell lines. In addition, chalcones inhibited the expression of AURKA and MDR-1 at the mRNA and protein level.

## 5. Material and Methods

### 5.1. Materials

The two tested compounds, *trans*-chalcone and Licochalcone A, and some of the main reagents including trypsin, doxorubicin, hydrochloride, dimethyl sulfoxide (DMSO), DMEM, penicillin, kanamycin, and streptomycin were purchased from Sigma-Aldrich (St. Louis, MO, USA). Fetal bovine serum was obtained from Cultilab (Campinas, SP, Brazil). The materials used for gene expression were *RNeasy^®^ Mini Kit*, *RNase-free DNase Set*, *RT^2^ SYBR^®^ Green JumpStart TAQ Ready Mix*, *RT^2^ First Strand Kit*, and *RT² Profiler™ PCR Array Human Cancer Drug Targets*. The BCA (Bicinchoninic Acid) protein assay was purchased from Thermo Scientific (Rockford, IL, USA).

### 5.2. Cell Culture

The breast cancer cell lines MCF-7 (Luminal A, estrogen receptor (ER) positive ER^+^/PR^+/−^/HER2) and BT-20 (Basal, triple negative-ER^−^/PR^−^/HER2^−^) were grown in DMEM medium supplemented with 10% fetal bovine serum, 100 U/mL penicillin, 100 μg/mL streptomycin, and 100 μg/mL kanamycin. The cultures were maintained in a humidified 5% CO_2_ atmosphere at 37 °C until 90% confluence.

### 5.3. Viability Assay Conditions

The inhibition of cell viability induced by *trans*-chalcone and Licochalcone A was evaluated by the MTT assay. This test was performed as described by Komoto et al. [28] with modifications. Briefly, each cell line (MCF-7 and BT-20) were seeded at a density of 2 × 10^3^ cell/well in 96-well plates and incubated for 24 h. The cells were treated with 7 different concentrations of the both chalcones for 24 h. Next, 20 µL MTT solution (5 mg/mL) was added to each well, and the plates were incubated for an additional 3 h. The formazan dye was solubilized in 200 µL DMSO. Absorbance was read at 550 nm in a microplate reader MultiSkan FC. The assays were carried out in three independent experiments performed in triplicate. Doxorubicin was used at 2.5 µg/mL (4.31 µM) as the positive control for this test. The results were plotted as the percentage of inhibition of cell viability (ICV) calculated as follows: ICV (%) = [1 − (absorbance of treated group/absorbance of control group)] × 100. Data represent the average ± SD from three independent experiments combined. The estimated averages were used to calculate the IC_50_ value by nonlinear regression.

### 5.4. RNA Extraction, cDNA Conversion and RT^2^ Profiler PCR Array

MCF-7 cells (at a density 2.5 × 10^4^ cells) were treated with IC_50_ value of chalcones for 24 h. The RNA extraction, cDNA conversion, and PCR array was performed as described by Bortolotto et al. [10]. The RT² Profiler™ PCR Array (Qiagen Hilden, Germany) was used to screen 84 genes related to Human Cancer Drug Targets. 

### 5.5. qRT-PCR

Besides of MCF-7 cell line we used the BT-20 cell line in this experiment in order to analyze the effect of chalcones on gene expression in the triple negative cell line. A set of 7 genes was used for qPCR assay (Table 2). These reactions were performed as described by Bitencourt et al. [29]. The GAPDH was used as the normalized gene, and the gene expression levels were calculated using the 2^−ΔΔCT^ comparative method. The results are reported as the mean ± standard deviation of three experiments.

### 5.6. Migration and Invasion Assays

Transwell assays (Corning^®^, Corning, NY, USA) were used to analyze the effect of *trans*-chalcone and licochalcone A on migration and invasion capacity of BT-20 cells, due to their high metastasis capacity. The assays were performed as described by Silva et al. [32]. The treatments were realized using 30 µM and 15 µM for 24 h. Briefly, cells were maintained in serum-free medium overnight, and then 2 × 10^6^ cells resuspended in 200 µL serum-free medium were seeded into the upper chamber of a 24-well Transwell plate with an 8 µm pore size (Corning^®^). Into the lower chamber, 750 µL of complete medium (with 10% serum) containing different concentrations of *trans*-chalcone or licochalcone A were added. After treatment for 24 h, the migrated cells attached on the bottom side of the Transwell. The membrane was fixed with 3.7% of paraformaldehyde for 2 min, permeabilized with methanol 100% for 20 min, and stained with Wright’s Giemsa solution for 15 min at room temperature. For the invasion assay, the membrane of the Transwell was pre-coated with 0.75 mm of Matrigel (Corning^®^) according to the manufacturer’s instructions, and then the same step detailed for migration assay was performed. Either for migration and invasion, the cells were destained with 100 µL of cells of 33% acetic acid. The destaining solution was collected and the absorbance was measured at a wavelength of 490 nm by a microplate reader (MultiSkan FC, Thermo Scientific, Waltham, MA, USA). The inhibition of migration or invasion was calculated as follows: Inhibition (%) = [1 − (absorbance of treated group/absorbance of control group)] × 100.

### 5.7. Western Blot Analysis

The Western Blot assay was performed to analyze the effect of chalcones on the modulation of MDR-1 and AURKA proteins. The cells MCF-7 and BT-20 were grown to 80% confluence in 60-mm dishes and treated with 40 or 80 µM of *trans*-chalcone and Licochalcone A in serum-containing medium for 24 h, according to the published protocols described by Silva et al. (2015) [16].

### 5.8. Statistical Analysis

All experimental data were submitted to an analysis of variance (ANOVA) followed by the Bonferroni test. The level of statistical significance was set at *p* < 0.05.

## Figures and Tables

**Table 1:** IC_50_ values (µM) of the two chalcones toward MCF-7 and BT-20 cell lines after 24 h treatments.

Cell Lines	*Trans*-Chalcone	Licochalcone A
MCF-7	53.73	60.46
BT-20	26.42	30.78

**Table 2:** Primers used in qPCR.

Genes	Sequences (5′–3′)	Amplicon Size	References
ABCG2	F: TTGATAGCCTCACCTTATTG	88	This paper
	R: ACCAGCTGATTCAAAGTATC		
ABCC1	F: AGCAGAAAAATGTGTTAGGG	176	This paper
	R: TACCCACTGGTAATACTTGG		
ABCC3	F: TTTTCTGGTGGTTCACAAAG	92	This paper
	R: GATCTGTCCTCTTCCTTTAG		
MDM4	F: AGATGAAACATCTAGGCTG	143	This paper
	R: CAATCCACCTGATTTGTCTG		
EGFR	F: GAAAAGAAAGTTTGCCAAG	195	This paper
	R: ATGAGGACATAACCAGCC		
AURKA	F: TACAAAAGAATATCACGGG	126	[30]
	R: AAGTACTTCTCTGAGCATTG		
Bcl-2	F: GATTGTGGCCTTCTTTGAC	164	This paper
	R: GTTCCACAAAGGCATCC		
GAPDH	F: ACAGTTGCCATGTAGACC	99	[31]
	R: TTTTTGGTTGAGCACAGG		

F: Forward R: Reverse.
